# A Short-Day Photoperiod Delays the Timing of Puberty in Female Mice *via* Changes in the Kisspeptin System

**DOI:** 10.3389/fendo.2018.00044

**Published:** 2018-02-20

**Authors:** Tabata Mariz Bohlen, Marina Augusto Silveira, Daniella do Carmo Buonfiglio, Hildebrando Candido Ferreira-Neto, José Cipolla-Neto, Jose Donato, Renata Frazao

**Affiliations:** ^1^Department of Anatomy, Institute of Biomedical Sciences, University of São Paulo, São Paulo, Brazil; ^2^Department of Physiology and Biophysics, Institute of Biomedical Sciences, University of São Paulo, São Paulo, Brazil; ^3^Department of Physiology, Augusta University, Augusta, GA, United States

**Keywords:** puberty onset, *Kiss1*, photoperiod, melatonin, energy balance

## Abstract

The reproduction of seasonal breeders is modulated by exposure to light in an interval of 24 h defined as photoperiod. The interruption of reproductive functions in seasonally breeding rodents is accompanied by the suppression of the *Kiss1* gene expression, which is known to be essential for reproduction. In non-seasonal male rodents, such as rats and mice, short-day photoperiod (SP) conditions or exogenous melatonin treatment also have anti-gonadotropic effects; however, whether photoperiod is able to modulate the puberty onset or *Kiss1* gene expression in mice is unknown. In the present study, we investigated whether photoperiodism influences the sexual maturation of female mice *via* changes in the kisspeptin system. We observed that SP condition delayed the timing of puberty in female mice, decreased the hypothalamic expression of genes related to the reproductive axis and reduced the number of *Kiss1*-expressing neurons in the rostral hypothalamus. However, SP also reduced the body weight gain during development and affected the expression of neuropeptides involved in the energy balance regulation. When body weight was recovered *via* a reduction in litter size, the timing of puberty in mice born and raised in SP was advanced and the effects in hypothalamic mRNA expression were reverted. These results suggest that the SP delays the timing of puberty in female mice *via* changes in the kisspeptin system, although the effects on hypothalamic–pituitary–gonadal axis are likely secondary to changes in body weight gain.

## Introduction

Photoperiod duration is one of the most potent environmental cues responsible for synchronizing daily variations in mammalian physiology, including factors that control the reproductive system. The mechanism by which the light/dark cycle synchronizes biological functions in mammals relies on the suprachiasmatic nuclei (SCN), which are considered the master clock that coordinates daily rhythms. SCN neurons receive information about the light/dark cycle *via* the retino-hypothalamic tract (1, 2). The lack of circadian rhythms, as induced by SCN ablation, or free-running rhythms, as induced by constant light, leads to reproductive deficits as demonstrated by impairment of ovulation, disruption of the estrous cycle and LH surge (3–5). Melatonin is the principal hormone produced by the pineal gland; its secretion is modulated by photoperiod duration, depends upon input from the SCN (6, 7), and is considered an important factor for the control of reproduction in melatonin-proficient mammals. Accordingly, estrous cyclicity can be reestablished by exogenous melatonin administration in female rats exposed to constant light (5). On the other hand, short-days or exogenous melatonin treatment has been described to induce anti-gonadotropic effects (5, 8–12). Nevertheless, the neuroendocrine mechanisms by which variations in photoperiod modulate the hypothalamus–pituitary–gonadal (HPG) axis are not fully understood.

Interestingly, the hypothalamic *Kiss1* expression is suppressed in winter or by artificial SP in Syrian hamsters, and this effect is prevented by ablation of the pineal gland (13, 14). Kisspeptins are encoded by the *Kiss1* gene and are known as the most potent activators of GnRH neurons (15, 16). Kisspeptin neurons in rodents are found in the anteroventral periventricular nucleus (AVPV), the rostral periventricular nucleus (PeN), and the arcuate nucleus of the hypothalamus (ARH) (13, 17, 18). However, whether photoperiod variations are able to directly affect kisspeptin neurons in mice remains unknown.

To investigate whether photoperiodism may influence the sexual maturation and the kisspeptin system of female mice, we bred mice expressing the humanized Renilla green fluorescent protein (hrGFP) under the transcriptional control of the *Kiss1* gene (19) and raised them under a short-day (6 h:18 h light/dark cycle) or a 12 h:12 h light/dark cycle. We assessed the timing of sexual maturation, and the data obtained were correlated with molecular analyses in the hypothalamus.

## Materials and Methods

### Animals

To evaluate the effects of photoperiod on the timing of puberty, we used the Kiss1-hrGFP mouse (C57BL/6-Tg(Kiss1-hrGFP)KG26Cfe/J, Jackson Laboratories) (19). This transgenic mouse model allows the visualization of *Kiss1*-expressing cells through the hrGFP protein. Mice were weaned at 3 weeks of age and genotyped *via* PCR using DNA extracted from the tail tip (REDExtract-N-Amp™ Tissue PCR Kit, Sigma). Kiss1-hrGFP mice were housed in the animal care facility of the Department of Anatomy, Institute of Biomedical Sciences, University of São Paulo, in an environment with controlled light and temperature (23 ± 2°C). All experiments and procedures were performed in accordance with the guidelines established by the National Institute of Health’s Guide for the Care and Use of Laboratory Animals and were approved by the Committee on the Care and Use of Laboratory Animals of the Institute of Biomedical Sciences, University of São Paulo.

### Evaluation of Sexual Maturation

The timing of puberty was investigated in pups born and raised at a constant 12 h:12 h light/dark cycle [control, lights on at 06:00 a.m.; Zeitgeber time (ZT) 0] or 6h:18 h light/dark cycle (SP, lights on at 12 p.m.). Kiss1-hrGFP mice were raised and bred in 12 h:12 h light/dark conditions and their offspring were included in the control group. A group of adult Kiss1-hrGFP mice were transferred to SP conditions at least 15 days before mating for acclimation. Only mice from average-sized litters (6–8 pups per litter) were included in control (*n* = 12) and SP groups (*n* = 14). To determine whether body mass gain would account for the differences in the timing of puberty in female mice born and raised in SP, we induced overnutrition by reducing the litter size to three pups at postnatal day 5, as previously described (20–22). Therefore, females were raised in small litters in SP (*n* = 13), and the obtained data were compared with data from females born and raised in regular litters (*n* = 16) in SP (6–8 pups per litter). Sexual maturation was determined by the age at vaginal opening, assessed through the first occurrence of vaginal cornification in the vaginal lavage (first estrus) and the onset of cyclicity, which is the first occurrence of an estrous cycle of typical duration (4–7 days in mice), as previously described (22, 23). These parameters were monitored daily, 1 h before lights off, until 70 days of age. Body weight was monitored weekly and at each specific stage of sexual maturation. The uterine mass was also determined in adult female mice (*n* = 4/10 per group).

### Relative Gene Expression

For gene expression analyses, 10- to 12-week old female mice in diestrus were selected after daily observation of vaginal smears and confirmation of a regular estrous cycle. Mice were sacrificed by decapitation in the morning (ZT2-3 for control group, *n* = 7; and ZT20-21 for SP group, regular litter, *n* = 7; small litter, *n* = 9). The hypothalamus was carefully dissected for relative gene expression analysis, according to previously described anatomical references (22). The total RNA from the hypothalamus was extracted with TRIzol Reagent according to the manufacturer’s instructions (Thermo Fisher Scientific). An assessment of RNA quantity was performed using an Epoch Microplate Spectrophotometer (BioTek). RNA was incubated in RNase-free DNase I (Roche Applied Science). Reverse transcription was performed with 2 µg of total RNA obtained from the hypothalamus with SuperScript II Reverse Transcriptase (Invitrogen) and random primers p(dN)6 (Roche Applied Science). Real-time polymerase chain reaction was performed using the 7500 Fast Real-Time PCR System (Applied Biosystems) and Power SYBR Green PCR Master Mix (Applied Biosystems). Specific primers were used for each target gene (Table 1) as previously described (22). Relative mRNA was quantified by calculating 2^−ΔΔ^Ct. Data were normalized to the geometric average of housekeeping genes *Actb, Gapdh*, and *Ppia* and reported as fold changes compared with values obtained from the control group (set at 1.0).

**Table 1:** Primer sequences.

Gene of interest	Accession no.	Forward primer (5′-3′)	Reverse primer (5′-3′)	Product size (bp)
*Actb*	NM_007393.5	AGCCTGGATGGCTACGTACA	CCTCTGAACCCTAAGGCCAA	90
*Agrp*	NM_001271806.1	CTTTGGCGGAGGTGCTAGAT	AGGACTCGTGCAGCCTTACAC	75
*Crh*	NM_205769.2	TGGATCTCACCTTCCACCTTCTG	CCGATAATCTCCATCAGTTTCCTG	103
*Ppia*	NM_008907.1	TATCTGCACTGCCAAGACTGAGT	CTTCTTGCTGGTCTTGCCATTCC	128
*Esr1*	NM_007956.5	GCAGATTGGGAGCAGCTGGTTCA	TGGAGATTCAAGTCCCCAAA	74
*Gal*	NM_010253	TGTCGCTAAATGATCTGTGGTTGTC	TGCAACCCTGTCAGCCACTC	121
*Gapdh*	NM_001289726.1	GGCAGCCCAGAACATCAT	CCGTTCAGCTCTGGGATGAC	75
*Gnrh1*	NM_008145.2	GGCTTCTGCCATTTGATCCAC	CCCTTTGACTTTCACATCC	200
*Kiss1*	NM_178260.3	GGCAAAAGTGAAGCCTGGAT	GATTCCTTTTCCCAGGCATT	75
*Npy*	NM_023456	CCCTCAGCCAGAATGCCCAA	CCGCCCGCCATGATGCTAGGTA	95
*Pomc*	NM_001278581.1	GAGGCCACTGAACATCTTTGTC	GCAGAGGCAAAACAAGATTGG	252
*Tac2*	NM_001199971	CCGCTCCATCTCTCTGGAAG	TGCATGTCACGTTTCTGTGG	94

### Brain Histology

To determine the number of *Kiss1*-expressing neurons in control (*n* = 4) and SP (*n* = 4) groups, we selected adult female mice in diestrus after daily observation of vaginal smears and confirmation of a regular estrous cycle. Animals were deeply anesthetized in the afternoon (ZT10-11 for control group and ZT4-5 for SP group), and perfused transcardially with saline followed by a 10% buffered formalin solution, pH 7.4. The brains were collected, post-fixed overnight at 4°C, and cryoprotected for 24–48 h at 4°C in 0.1 M phosphate-buffered saline (PBS) containing 20% sucrose, pH 7.4. Brains were cut in four series of 30 μm sections each in the frontal plane on a freezing microtome and stored at −20°C in cryoprotectant, until they were processed for immunohistochemistry to detect hrGFP-immunoreactivity.

Brain sections were rinsed in 0.02 M potassium PBS (KPBS), pH 7.4, followed by a pretreatment with 0.3% hydrogen peroxide for 30 min. After rinses in KPBS, the sections were blocked in 3% normal donkey serum for 1 h, followed by incubation in anti-hrGFP antibody (1:2,000, Stratagene) for 24 h. Subsequently, the sections were incubated with biotin-conjugated donkey anti-rabbit IgG (1:1,000, Jackson Laboratories) for 1 h followed by an avidin–biotin complex (1:500, Vector Labs, Burlingame) for 1 h. The peroxidase reaction was performed using 0.05% 3,3′-diaminobenzidine and 0.03% hydrogen peroxide. The sections were mounted onto gelatin-coated slides and coverslipped using DPX mounting medium.

The number of *Kiss1*-expressing neurons were quantified in two rostrocaudal levels of the AVPV/PeN (relative to bregma: 0.26 and 0.02) and two rostrocaudal levels of the ARH (relative to bregma: −1.94 and −2.30). The approximate bregma coordinates of each rostrocaudal level were obtained from the mouse brain atlas (24). The cells were counted on one side of a defined level of each nucleus, as previously described (19). The photomicrographs of brain sections were acquired using a Zeiss Axiocam HRc camera connected to a Zeiss AxioImager A1 microscope (Zeiss, Munich, Germany), and the images were digitized using AxioVision software (Zeiss). Photoshop image-editing software was used to combine the photomicrographs into plates. Only the sharpness, contrast and brightness were adjusted.

### Statistical Analysis

Statistical analyses were performed using GraphPad Prism software. The data were expressed as the mean ± SEM. The comparisons between two groups were performed using the unpaired two-tailed Student’s *t*-test. For temporal body weight analyses, we used the two-way ANOVA and the Bonferroni post-test. A *P* value <0.05 was considered statistically significant.

## Results

### A Short-Day Photoperiod (SP) Delays the Sexual Maturation of Female Mice

**Figure 1:**
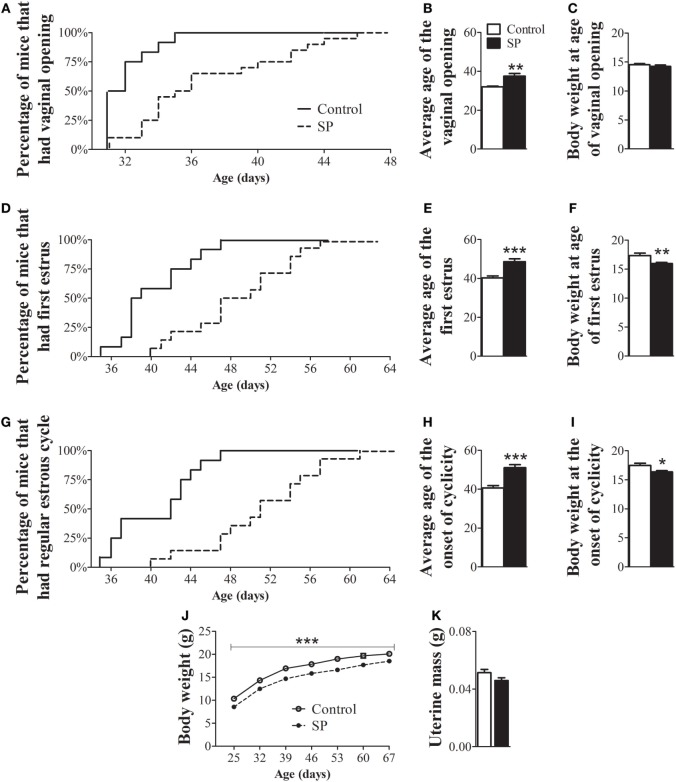
Female mice born and raised under a short-day photoperiod (SP) exhibit delayed sexual maturation. **(A,D,G)** Graphs showing the percentages of control females and females born and raised under a SP (*n* = 12/14 per group) that exhibited vaginal opening **(A)**, first estrus **(D)**, and the first occurrence of an estrous cycle of typical duration **(G)**. **(B,E,H)** Average time required for control and SP females to exhibit vaginal opening **(B)**, first estrus **(E)**, and the onset of cyclicity **(H)**. **(C,F,I,J)**. Body weight of control and females under SP at specific stages of sexual maturation and throughout development **(J)**. **(K)** Uterine mass of adult controls and adult females in SP (*n* = 9/10 per group). *P < 0.05, **P < 0.005, ***P < 0.0005 vs control.

To evaluate whether SP influences the timing of puberty in a non-seasonal breeder, we assessed the age of vaginal opening, first estrus and onset of estrus cyclicity in female mice. Females raised in SP exhibited a significant delay in the age of vaginal opening (Figures 1A,B; *P* = 0.0013), first estrus (Figures 1D,E; *P* = 0.0001) and the onset of estrous cyclicity (Figures 1G,H; *P* < 0.0001) compared to control female mice. While more than 80% of control mice had displayed vaginal opening by 33 days of age, only 25% of females of the SP group had reached the same stage of sexual maturation (Figure 1A). Approximately 80% of control females had their first estrus by 44 days of age, whereas less than 25% SP females reached estrus by that age (Figure 1D). In addition, while 100% of control females had already shown regular estrous cycles by 47 days of age, less than 30% of females under SP had started to cycle at the same age (Figure 1G). At the age of vaginal opening, female mice raised in SP exhibited similar body weight to control females (Figure 1C; *P* = 0.39). However, at the ages of first estrus (Figure 1F; *P* = 0.005) and onset of cornification (Figure 1I; *P* = 0.01), females under SP exhibited lower body weight compared to controls. Because photoperiodism has been previously shown to influence the body weight of seasonally and non-seasonally breeding rodents (11, 12, 25, 26), we evaluated in more detail whether the SP could affect the body weight gain during development. Our findings showed that mice born and raised in SP exhibited a lower body weight gain throughout development (Figure 1J; *P* = 0.0005). In addition, no significant differences between groups were noted in the uterine mass of adult mice (Figure 1K, *P* = 0.08).

### A SP Modulates the Hypothalamic Expression of Genes Related to the Reproductive Axis and Energy Balance

**Figure 2:**
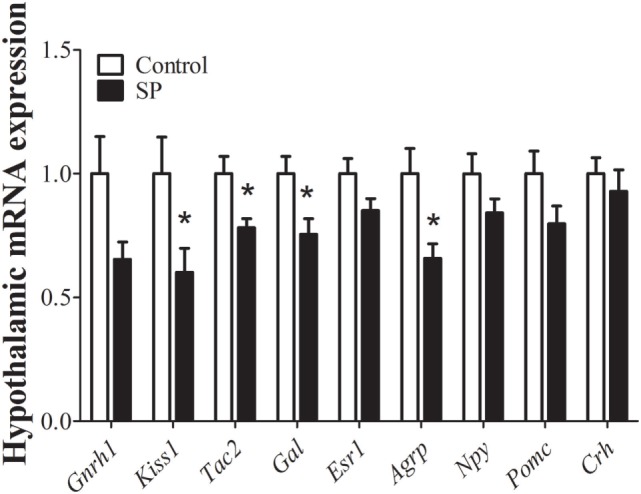
Hypothalamic mRNA expression analysis in control animals and in females born and raised under a short-day photoperiod (SP; *n* = 7 per group). *P < 0.05 vs control.

Because neurons responsible for coordinating reproductive function and energy balance are distributed in the hypothalamus, we investigated whether the delayed sexual maturation and reduced body weight of animals raised under SP were accompanied by changes in hypothalamic gene expression. We first assessed the expression of genes related to the reproductive axis. Interestingly, adult females raised under SP showed decreased expression of the mRNA encoding the *Kiss1* gene (*P* = 0.04), Tachykinin (*Tac2, P* = 0.02), and Galanin (*Gal, P* = 0.02) and exhibited a trend toward lower expression of mRNA encoding GnRH (*Gnrh1, P* = 0.06) compared to control mice (Figure 2). No significant changes between groups were observed in the hypothalamic mRNA encoding the estrogen receptor alpha (*Esr1*; *P* = 0.1) (Figure 2). In addition, because females raised under SP were leaner, we also assessed the hypothalamic mRNA expression of neuropeptides involved in the regulation of energy balance, such as agouti-related protein (A*grp*), neuropeptide Y (*Npy*), and pro-opiomelanocortin (*Pomc*). *Agrp* mRNA expression was significantly decreased in the hypothalamus of females born and raised under SP compared with control females (*P* = 0.01; Figure 2). No changes in *Npy* (*P* = 0.13) or *Pomc* (*P* = 0.13) mRNA expression were observed. In addition, the expression of corticotropin-releasing hormone (*Crh*) mRNA, which could indicate a stress-mediated response, was not influenced by the short-day photoperiod (*P* = 0.5; Figure 2).

### Photoperiod Leads to a Reduced Number of *Kiss1*-expressing Neurons

**Figure 3:**
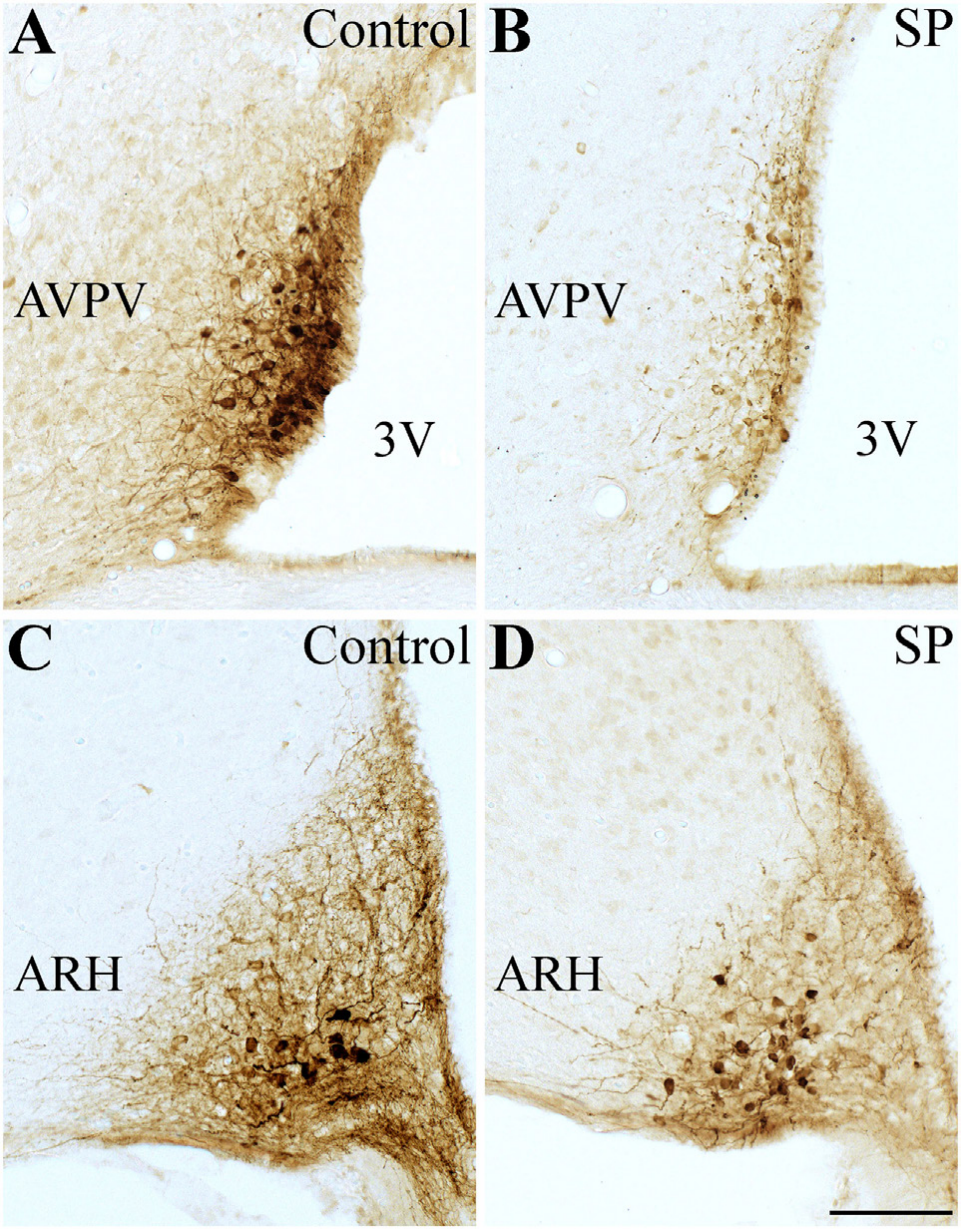
Effects of the short-day photoperiod (SP) on kisspeptin hypothalamic expression. **(A–D)** Brightfield photomicrographs of mouse brain sections showing the expression of the humanized Renilla green fluorescent protein (hrGFP) in the anteroventral periventricular nucleus [AVPV **(A,B)**] and in the arcuate nucleus [ARH **(C,D)**]. **(B,D)** SP led to a reduced number of *Kiss1*-expressing neurons in the AVPV compared to control **(A,C)**. Abbreviation: 3 V, third ventricle. Scale bar: 50 µm.

We used Kiss1-hrGFP mice to determine the number of *Kiss1*-expressing cells, as previously described (19). Interestingly, females raised under SP had lower number of neurons expressing hrGFP compared with control mice (Figure 3). In the AVPV/PeN, we observed a reduction of approximately 50% in the number of hrGFP cells compared with control mice (Control: 29.7 ± 2.3 cells; SP: 15.8 ± 2.3 cells; *n* = 4, *P* = 0.002). However, the number of hrGFP cells in the ARH was similar between the groups (Control: 20.1 ± 4.1 cells; SP: 14.0 ± 1.4 cells; *n* = 4, *P* = 0.2). These results indicate that the delayed sexual maturation observed in SP females could be caused by lower kisspeptin expression in the AVPV/PeN.

### The Delayed Puberty Timing Caused by SP Depends on Changes in Body Weight

**Figure 4:**
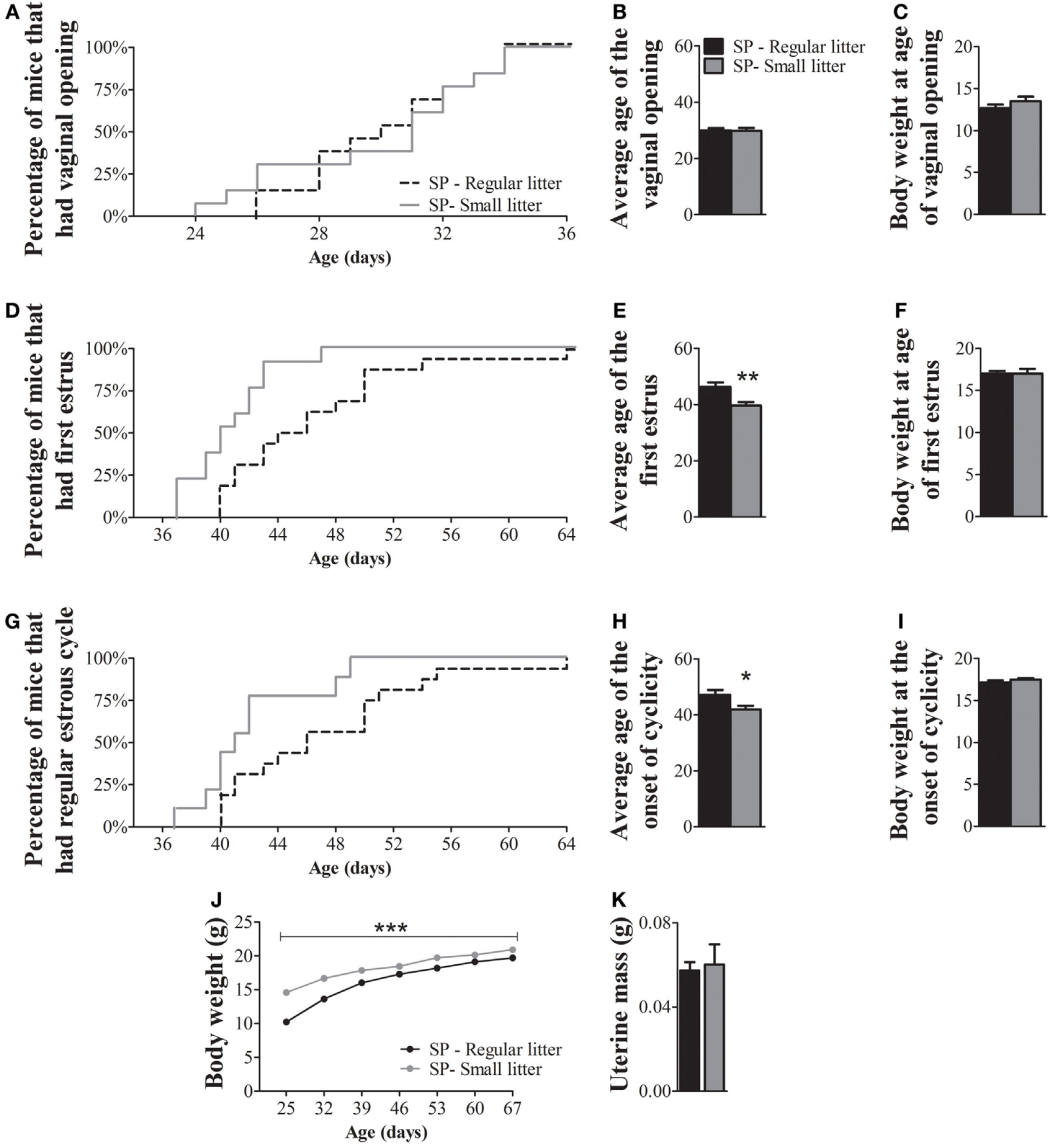
Increased body weight advances the timing of puberty in female mice born and raised under a short-day photoperiod (SP). **(A,D,G)** Graphs showing the percentages of females from regular control litters compared to females from small litters that exhibited vaginal opening **(A)**, first estrus **(D)**, and the first occurrence of an estrous cycle of typical duration [**(G)** *n* = 13/16 per group]. **(B,E,H)** Average time required for females to exhibit vaginal opening **(B)**, first estrus **(E)**, and the onset of estrous cyclicity **(H)**. **(C,F,I,J)**. Body weight of females from regular and small litters at specific stages of sexual maturation and during development **(J)**. **(K)** Uterine mass of adult females from regular litters and small litters (*n* = 4/6 per group). *P < 0.05, **P < 0.005 vs females from regular control litter.

Since nutritional status is known to affect the timing of puberty (22, 27–30), we next evaluated whether a protocol that increases body mass could revert the observed effects in the puberty timing of females born and raised under SP. We observed that females born under SP and raised in small litters exhibited vaginal opening at a similar age as females born under SP and raised in regular litters (Figures 4A,B; *P* = 0.9). However, the average age of the first estrus (Figures 4D,E; *P* = 0.004) and the onset of estrous cyclicity (Figures 4G,H; *P* = 0.04) of females from small litters were significantly advanced compared to those of females from regular litters. While more than 70% of females from small litters had their first estrus by 42 days of age, approximately 30% of females from regular litters had reached the same stage of sexual maturation at that age (Figure 4D). At 42 days of age, more than 70% of females from small litters had started to cycle, compared with approximately 30% of females from regular litters under SP (Figure 4G). Although no significant differences in body weight were observed between groups at any specific stage of sexual maturation (Figures 4C,F,I), females from small litters exhibited larger body weight than females from regular litters during development (Figure 4J; *P* < 0.0001). In addition, no significant differences were noted in the uterine mass of adult mice (Figure 4K; *P* = 0.7).

**Figure 5:**
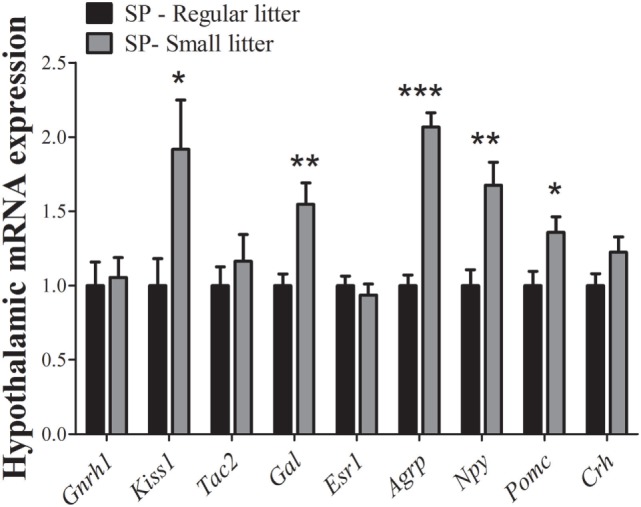
Hypothalamic mRNA expression analysis comparing data obtained of females from regular control or small litters born and raised under a short-day photoperiod (SP, *n* = 7/9 per group). *P < 0.05, **P < 0.005, ***P < 0.0001 vs regular litter.

Given that the effects on the timing of puberty were probably dependent on body weight changes, a reversal in hypothalamic mRNA expression should be expected in SP females raised in small litters. In fact, we observed that hypothalamic *Kiss1* mRNA (*P* = 0.04) and *Gal* mRNA expression levels (*P* = 0.008) were significantly increased in females from small litters compared with females from regular litters under SP. No significant differences were noted in *Gnrh1* (*P* = 0.8), *Tac2* (*P* = 0.5), *Esr1* (*P* = 0.5), or *Crh* mRNA expression (*P* = 0.1, Figure 5). In addition, significant increases in the hypothalamic mRNA expression levels of *Agrp* (*P* < 0.0001), *Npy* (*P* = 0.005), and *Pomc* (*P* = 0.03) were observed in females from small litters born and raised in SP (Figure 5).

## Discussion

In the present study, we used a SP condition to demonstrate how photoperiodic information affects the timing of puberty in female mice and whether these effects occur *via* kisspeptin system. Since we observed that SP also affected energy homeostasis, we also tested the hypothesis that photoperiodism may regulate the timing of sexual maturation in female mice *via* changes in body weight.

In seasonally breeding rodents, the effects of short-photoperiod in the reproductive system are attributed to an extended period of melatonin secretion (18). Importantly, although several reports demonstrated that most mouse strains are melatonin deficient (31–33), others have reported that some strains, such as C57BL/6, are able to produce small amounts of melatonin (34, 35). Therefore, C57BL/6 strain has been routinely and successfully used in melatonin studies (36, 37). Accordingly, SP was able to cause robust changes in our mouse model, which indicates that C57BL/6 mice are clearly responsive to photoperiod variations. The observed effects on sexual maturation may be time-dependent, since C57BL/6 mice are unable to mount a photoperiodic response after only 2 weeks of acclimation in SP (38).

Previous studies that evaluated the importance of photoperiod or melatonin in reproduction showed no information about body weight changes (13, 14, 39). However, the effects of SP in reducing the body weight of seasonal rodents have been documented and these changes can be prevented by the removal of the pineal gland (26, 40, 41). The observed effects on body weight in the present work were obtained independently of environmental conditions, such as food availability, suggesting that photoperiod directly modulates the energy balance of female mice, as previous described (11). In rats and mice, melatonin is a known metabolic factor capable of reducing fat mass and circulating leptin levels; two well-known metabolic cues that determine the timing of sexual development (12, 22, 27–30, 42–44). Therefore, although SP could directly regulate the timing of sexual maturation, our results suggest that fat mass deposition can also be an important factor that influences the timing of puberty in female mice. To test the hypothesis that body weight gain was the most critical factor required for sexual maturation, we used a model of prepubertal overnutrition by raising mice in small litters in SP (20–22). The increased body weight of females raised in small litters and SP advanced the timing of puberty, as demonstrated by the early age of detection of first estrus and the onset of estrous cyclicity compared with females raised in regular litters and SP. Previous studies have already demonstrated that the small litter protocol is efficient in causing prepubertal overnutrition, increasing body weight and advancing markers of sexual maturation (20–22, 44).

Here, we demonstrated that, additionally to the lower body weight, mice raised in SP exhibited a significant reduction of *Kiss1* mRNA hypothalamic levels, compared to mice raised in 12 h:12 h light/dark cycle, similar to what has been described for hamsters (13, 14). In addition, *Tac2* and *Gal* mRNA expression levels were also suppressed by SP in female mice. Histological experiments further determined that the decrease in *Kiss1* expression mainly depended on a reduced number of *Kiss1*-expressing neurons in the AVPV/PeN, whereas the number of *Kiss1*-expressing cells was similar in the ARH of control and SP mice, consistent with what has been previously reported in rats (13). In contrast to our results, hamsters maintained under SP show lower *Kiss1* mRNA expression in both the ARH and the AVPV/PeN (14). These differences may indicate interspecies variations. Regardless, it is important to remember that undernutrition, as induced by fasting, is sufficient to reduce *Kiss1* mRNA expression in rats (45, 46), which supports the hypothesis that the observed effects of SP on the timing of puberty are dependent on energy stores. In agreement with this idea, we observed that female mice raised in small litters in SP exhibited increased *Kiss1* and *Gal* mRNA expression levels. In addition, SP clearly affected the expression of genes known to modulate energy balance in mice raised in both regular and small litters. Female mice born and raised under SP exhibited decreased expression of *Agrp* mRNA compared to mice raised in 12 h:12 h light/dark cycle. Since AgRP has orexigenic effects, these changes could help to explain the leaner body weight observed in this group. On the other hand, mice from small litters and raised under SP showed significant increases in *Agrp* and *Npy* mRNA expression in the hypothalamus, indicating that the orexigenic effects of these neuropeptides could be responsible for the higher body mass gain in this group. By contrast, *Pomc* encodes anorexigenic neuropeptides, including α-MSH. Although hypothalamic *Pomc* expression normally exhibits the opposite behavior compared to *Agrp*, in our experiments *Pomc* levels varied similarly to *Agrp/Npy* expression. It is possible that the changes observed in *Pomc* expression are an attempt to compensate for the increased *Agrp/Npy* expression and, consequently, to restore energy homeostasis. Previous studies have shown that hypothalamic expression of *Pomc* is increased in situations that induce obesity, such as a high-fat diet or ovariectomy (47, 48). Altogether, these findings provide evidence that SP induces changes in body weight *via* hypothalamic neurocircuits that regulate energy balance. Therefore, future studies could investigate the mechanisms or factors recruited by SP to affect the expression of key neuropeptides that regulate energy homeostasis, especially in *Agrp/Npy* expressing neurons. In addition, we cannot rule out the hypothesis that the results obtained were dependent on circadian time because tissue collection from mice raised in 12 h:12 h light/dark and in SP were collected at different ZT. However, as demonstrated previously (49), sacrifice ZT did not affect gene expression of peptides related to the reproductive axis since all females were in diestrus. Considering that there is evidence in humans that reproductive dysfunctions, such as precocious puberty or functional hypogonadotropic hypogonadism, may be followed by significant variations in melatonin levels (50–53), future studies should determine whether such disorders were accompanied by changes in energy homeostasis.

In summary, our results demonstrate that SP affects the timing of puberty in female mice. The effects in sexual maturation are followed by a decrease in body weight gain during development and by a lower *Kiss1* mRNA expression in the hypothalamus. Reestablishment of body weight gain in SP conditions by reduction of litter size leads to advanced timing of puberty and increased *Kiss1* mRNA levels, indicating that the effects of SP in the HPG axis are likely secondary to changes in body weight gain.

## Ethics Statement

All experiments and procedures were performed in accordance with the guidelines established by the National Institute of Health’s Guide for the Care and Use of Laboratory Animals and were approved by the Committee on the Care and Use of Laboratory Animals of the Institute of Biomedical Sciences, University of São Paulo.

## Author Contributions

TMB, MAS, DCB and HCFN performed the experiments. RF, JDJ and JCN designed the experiments. RF coordinated the studies and wrote the paper.

## Conflict of Interest Statement

The authors declare that the research was conducted in the absence of any commercial or financial relationships that could be construed as a potential conflict of interest.
